# Perceptions, Practices, and Prevalence of Nursing Peer Review Among Nurse Managers in Qatar: A Cross-Sectional Study

**DOI:** 10.1155/jonm/8854800

**Published:** 2025-07-28

**Authors:** Fadi Amro, Ahmad Rayan, Fadwa Alhalaiqa, Mohammed Aldalaykeh, Ali Aldirawi, Nidal Eshah, Ahmad Rayani

**Affiliations:** ^1^Faculty of Nursing, Zarqa University, Zarqa, Jordan; ^2^College of Nursing, Qatar University, P.O. Box 2713, Doha, Qatar; ^3^Xiangya School of Nursing, Central South University, Changsha, Hunan, China; ^4^Community and Psychiatric Mental Health Nursing Department, College of Nursing, King Saud University, Riyadh 12371, Saudi Arabia

**Keywords:** nurse managers, nursing, peer review, perception, performance appraisals, prevalence, work incidents

## Abstract

**Background:** Nursing peer review (NPR) is a critical process for improving nursing practices through timely feedback, fostering accountability, and enhancing care quality. Effective integration of NPR strategies in healthcare organizations requires an understanding of its perceptions, practices, and prevalence among nursing leaders, which serves as the foundation for successful implementation and sustainability.

**Aim:** To evaluate NPR perceptions, practices, and prevalence among nurse managers in Qatar.

**Methods:** This descriptive, cross-sectional study surveyed 244 nurse managers at Hamad Medical Corporation (HMC) in Qatar. Data were collected using a validated tool that encompasses the NPR structure, process, prevalence, and perception metrics. Statistical analyses were performed using SPSS software. This study used STrengthening the Reporting of OBservational studies in Epidemiology (STROBE) guidelines for reporting.

**Results:** NPR processes demonstrated widespread adoption, with 85.2% of respondents reporting nursing-led committees and 94.7% indicating the presence of unit-based councils (UBCs). NPR was prevalent at both unit and organizational levels, with 91.4% of participants identifying it as a necessary process. Magnet-seeking organizations reported higher engagement in NPR activities, with 42.2% citing nurse-to-nurse handoff/shift reports as a frequent NPR activity. Significant associations were observed between the magnet status and the availability of nursing-led structures (75.4%, *p*=0.005) and NPR necessity (80.3%, *p*=0.004). Moreover, 88.3% of participants viewed NPR as integral to quality and safety efforts, and 93% recognized its role in fostering professional autonomy and accountability.

**Conclusions:** Nurse managers expressed positive perceptions of NPR, recognizing its role in improving clinical practice and care quality. The findings highlight the association between NPR and magnet-seeking efforts, underscoring the importance of aligning NPR policies with American Nursing Association (ANA) guidelines and magnet criteria to enhance implementation and sustainability.

## 1. Introduction

Nurses constitute the largest occupational workforce within healthcare organizations, playing a pivotal role in delivering high-quality care across diverse settings. Their responsibilities significantly contribute to the operational efficiency and cost structures of hospitals [1]. Through their daily practices, nurses are instrumental in ensuring high-quality care delivery [2]. Nursing peer review (NPR) has emerged as a valuable approach to maintain and enhance these standards. In this study, the peer review process refers specifically to formal and informal evaluations conducted by nurses on their peers' clinical performance, aligned with American Nurses Association (ANA) guidelines, with the goal of improving practice quality, accountability, and professional development. NPR offers an opportunity for professional development and improved health outcomes by fostering structured feedback and evaluation mechanisms [3]. Specifically, peer review practices among healthcare professionals, including nurses, aim to assess and enhance clinical practices by providing timely and constructive feedback [4, 5]. NPR practices are particularly focused on promoting patient care quality while supporting nurses' continuous professional development [6]. Moreover, NPR plays an essential role in performance evaluation, professional growth, and goal setting [7].

In healthcare, peer review is broadly defined as “a process whereby a committee evaluates the quality of professionals' clinical work to ensure that prevailing standards of care are being met” [8]. The ANA first defined NPR in 1988 as “an organized effort whereby practicing professionals review the quality and appropriateness of services ordered or performed by their peers.” This definition remains relevant today, emphasizing NPR's role in fostering accountability, autonomy, and a culture of continuous learning within the nursing profession [9]. NPR encompasses various forms and processes, including case reviews, chart audits, performance evaluations, self-assessments, and continuing education activities. These processes serve specific purposes, from reflecting on clinical practices and identifying areas for improvement to supporting professional development [10]. For instance, incident-based peer reviews, aligned with ANA guidelines, are often conducted after critical events to ensure adherence to quality and safety standards [11, 12].

NPR facilitates the sharing of best practices, fosters personal and professional development, and provides a framework to address variations in nursing care. By engaging direct-care nurses in peer evaluations, NPR encourages participatory approaches to identifying and mitigating potential near-misses or healthcare-related incidents [13]. It also enhances governance, professionalism, and accountability within the nursing profession, thereby advancing organizational outcomes and practice standards [6, 14]. Despite its proven benefits, NPR remains inconsistently implemented and understood across healthcare settings. Studies have revealed substantial variability in NPR prevalence, with rates ranging from 7% to 60% depending on the type of NPR conducted [9]. Confusion between NPR and performance evaluations further complicates its implementation, particularly at the unit level, where the core principles of timeliness, peer input, and development-focused feedback are sometimes overlooked [15].

Nurse managers play a critical role in integrating NPR within healthcare organizations. They are responsible for establishing structured feedback mechanisms, aligning NPR activities with organizational goals, and creating supportive environments for peer review. These responsibilities include resource management, staff training promotion, and adherence to NPR principles [16]. However, limited research has examined nurse managers' perceptions of NPR or their involvement in implementing NPR practices, particularly in the context of Qatar and the broader Middle Eastern region. Most existing literature originates from Western healthcare settings, which may differ significantly in the organizational structure, cultural dynamics, and leadership approaches. This study uniquely addresses this gap by providing empirical data on NPR implementation in a Middle Eastern country, offering insights that can inform culturally relevant policy development and leadership strategies in similar healthcare systems. While NPR has been shown to enhance accountability, professional development, and patient care outcomes [17–19], its adoption and effectiveness in Qatar remain largely unexplored. Whitney et al. emphasized that NPR is often underutilized due to unclear perceptions and inconsistent practices [9].

Building on existing studies, this research seeks to address the gaps in NPR implementation by exploring nurse managers' perceptions and practices in Qatar. Understanding these factors is critical for developing targeted interventions that promote NPR and improve nursing outcomes at both unit and organizational levels, especially in achieving organizational goals such as magnet certification (an international recognition awarded to healthcare organizations demonstrating nursing excellence and innovation in professional practice), which remains underexplored. Organizations actively working toward this designation are referred to as ‘Magnet-seeking.'

## 2. Materials and Methods

### 2.1. Study Design and Population

This study utilized a descriptive, cross-sectional correlational design to investigate NPR practices, perceptions, and prevalence among nurse managers in Qatar. The target population included nurse managers employed in various healthcare facilities under Hamad Medical Corporation (HMC), the primary healthcare provider in Qatar. The accessible population consisted of nurse managers holding management roles across HMC hospitals and departments, ensuring a diverse representation of expertise. Nurse managers were defined as registered nurses with titles such as chief nursing executive, assistant chief nursing executive, nursing director, head nurse, nursing in-house supervisor, or those acting in similar managerial capacities.

### 2.2. Study Settings

Data collection occurred within HMC hospitals, the primary healthcare provider in Qatar, representing approximately 90% of acute care services. Hospitals in Doha, Al Wakrah, and Al Khor cities served as the primary data collection sites, representing a wide range of nursing departments and facilities. This study used STrengthening the Reporting of OBservational studies in Epidemiology (STROBE) guidelines for reporting.

### 2.3. Sampling Method and Sample Size

A convenience sampling method was used to recruit participants, leveraging the most readily available population of nurse managers. The required sample size was calculated using a standard formula for a finite population correction. Assuming a 50% prevalence rate for NPR practices, a 95% confidence interval, and a 5% relative precision, a total of 400 active nurse managers were identified. Accounting for a 5% nonresponse rate, the minimum required sample size was 196. Ultimately, 244 participants completed the survey, yielding a 60% response rate. Eligible participants met the following criteria: Nurse managers with active roles, as designated by the HMC human resource system; holders of a Bachelor of Science in Nursing (BScN) degree; male and female nurse managers of all nationalities; English-speaking (not limited to native speakers); and no restrictions on years of experience in nursing management. Meanwhile, we excluded nurses who did not hold the title or responsibilities of a nurse manager and individuals not currently assuming nursing management roles.

### 2.4. Measurement Tool

The survey tool developed by Whitney et al. [9] was used to assess NPR perceptions, practices, and prevalence rates. The instrument was originally designed in collaboration with a panel of chief nurse executives (CNEs) and subject matter experts to ensure content validity. This validated tool comprises three main sections.

• Demographic questions: 13 items covering age, gender, years of experience, education level, and organizational details.
• NPR practices and prevalence: 10 questions describing current NPR practices and forms, including “check all that apply” responses.
• Perception questions: Ten Likert-scale questions plus one binary item. The perception items are scored from 1 (strongly disagree) to 5 (strongly agree), producing a total score range from 10 to 50, with higher scores indicating more favorable perceptions of NPR. Regarding the tool's validity and reliability, Whitney et al. emphasize that the NPR tool is considered valid as it is grounded in evidence from the literature [9]. Whitney et al. reported high content validity through expert panel review and pilot testing in U.S. healthcare institutions. Internal consistency reliability of the perception subscale was established with a Cronbach's alpha of 0.84 in the original study. In the current study, face validity was evaluated by five CNE-level experts and NPR specialists, while two experts further tested the survey's validity prior to its implementation. A pilot study involving eight nurse managers from various hospitals was conducted to assess the survey tool's clarity, feasibility, and reliability, yielding a Cronbach's alpha of 0.86. Feedback from the pilot study confirmed the appropriateness of the tool, with no modifications deemed necessary. Notably, responses from the pilot study were excluded from the final analysis to maintain the integrity of the study findings.

### 2.5. Data Collection and Ethical Considerations

This study adhered to declaration of Helsinki 1964. Ethical approval was obtained from the Institutional Review Board (IRB) at Zarqa University and HMC's Medical Research Center (MRC). Following approvals, eligible nurse managers were identified through HMC's central workforce departments. Data collection was conducted via email invitations, including a survey link, and supplemented by social group discussions, bulletin announcements, and individual communications. The data collection period lasted 6 months. Participation was voluntary, with full anonymity and confidentiality maintained. Participants were informed of their right to withdraw at any stage without consequences. Employment identifiers were not collected, and data were securely stored, with HMC retaining the right to review the findings.

### 2.6. Statistical Analysis

Data were analyzed using SPSS version 26. Data were screened for missing values, outliers, normal distribution, and assumptions before the actual statistical analysis. Descriptive statistics were used to summarize the sample characteristics and NPR prevalence rates. The chi-square test was used to examine the association between NPR practices and prevalence and magnet certification status.

## 3. Results

### 3.1. Sociodemographic Characterization

The study sample included 244 nurse managers, with a response rate of approximately 60%. The sample included slightly more male nurse managers (*n* = 152, 62.3%) than female nurse managers, with two-thirds of participants aged between 35 and 45 years. Approximately, 42% of the nurse managers reported having a master's degree. In terms of professional experience, two-thirds of participants (*n* = 161) reported having 10–20 years of nursing experience. However, most nurse managers had less than 10 years of management experience (*n* = 177, 72.5%). Head nurses represented the largest group by role (*n* = 143, 58.6%), followed by nursing house supervisors (*n* = 35, 14.3%). Regarding their workplace, one-third of participants were working at hospitals (*n* = 83, 34%), and one-third of participants were working at tertiary hospitals (*n* = 82, 33.6%). Approximately, one-third of managers were employed in inpatient departments. Most nurse managers (85.7%) expressed intent to seek magnet certification (See Table 1).

### 3.2. NPR Practices

The majority of nurse managers reported the availability of nursing-led committees or councils (*n* = 208, 85.2%), shared governance strategies (*n* = 193, 79.1%), and unit-based councils (UBCs) (*n* = 231, 94.7%) within their organizations. UBCs, which are formal groups of clinical nurses who collaborate on decisions affecting unit practice and patient care, are a key component of shared governance structures supporting NPR. NPR was reported as a formal process in 76.2% of participants' organizations, with 91.4% indicating that NPR was mandatory for all or specific nursing levels. However, for 53.7% of participants, NPR participation was limited to peer review committees, and 32% reported that NPR was implemented only in selected few units. Notably, 14.3% of participants stated that NPR was not practiced at all in their organizations. Education on providing constructive peer-to-peer feedback varied significantly. Over half of the respondents (52.9%) indicated that this education occurred quarterly or monthly, while 25.8% reported that it was conducted annually. Alarmingly, 21.3% of nurse managers indicated that no such education took place in their units. Regarding the integration of NPR into the annual performance evaluation process, feedback was predominantly delivered by managers (*n* = 187, 76.6%), with peers less frequently involved (*n* = 161, 66%). Furthermore, only 59% (*n* = 144) of participants reported that NPR feedback was formally associated with the annual evaluation process. Anonymity in NPR practices was reported by 29.9% (*n* = 73) of respondents, while 70.1% (*n* = 171) indicated that the process was not anonymous. Challenges to effective NPR implementation were also highlighted, with participants noting limited resources to support NPR activities (*n* = 122, 50%), insufficient consideration of nursing experience levels (*n* = 134, 54.9%), and infrequent opportunities for nurses to provide feedback about practice concerns (*n* = 132, 54.1%).  Table 2 presents the distribution of reported NPR practices among nurse managers, including committee structures, participation levels, and feedback mechanisms (see Table 2).

The most frequently reported NPR activity at the unit level was nurse-to-nurse handoff/shift reports (*n* = 103, 42.2%), followed by self-reported case reviews (*n* = 87, 35.7%). At the organizational level, incident-based/risk-based peer review committees (*n* = 82, 33.6%) and peer review for clinical ladder advancement (*n* = 82, 33.6%) were the most common forms. Across both levels, annual performance evaluations conducted by managers were the most prevalent NPR activity (*n* = 107, 43.9%), while self-reported case reviews were the least frequently reported across both levels.

The prevalence of NPR activities varied significantly between unit and organizational levels. For example, new evidence-based practice reviews were reported by 29.5% (*n* = 72) of participants at the unit level, compared to 25.4% (*n* = 62) at the organizational level, with 41% (*n* = 100) reporting prevalence across both levels. Similarly, peer competency skill assessments and peer interviewing for nursing roles were equally reported at both levels (36.1%, *n* = 88 each). The least reported NPR activities were focused individual quality reviews at the unit level (21.3%, *n* = 52) and annual performance evaluations by peers at the organizational level (24.2%, *n* = 59).

These findings highlight the variation in NPR practices between unit and organizational contexts, suggesting opportunities for standardizing NPR activities to enhance consistency and impact (see Table 3).

The majority of managers endorsed that NPR advances clinical practice from novice to expert (94.2%) and supports professional autonomy and accountability (93%). Most managers perceived NPR as an integral process for improving quality and safety (88.3%) and believed that constructive peer feedback was moderate to extremely important (80%). On the other hand, only 62.7% of managers reported that providing and receiving peer-to-peer feedback were relatively easy, and half of the sample reported difficulty in implementing NPR processes in their facilities (see Table 4).

The chi-square analysis revealed significant associations between the magnet-seeking status and several aspects of NPR practices. Magnet-seeking organizations were more likely to require NPR at all or some levels, with 80.3% of managers from these organizations indicating that NPR was needed, compared to 11.1% of managers from nonmagnet-seeking organizations (*p*=0.004). Conversely, 5.3% of magnet-seeking managers stated that NPR was not required, compared to 3.3% of those in nonmagnet-seeking organizations (*p*=0.041). Among magnet-seeking organizations, 75% clarified that NPR participation was often limited to committee members or specific units, highlighting variability in NPR implementation (*p*=0.041). Further, a significant association was found between the magnet-seeking status and the presence of nursing-led committees and councils. Among magnet-seeking organizations, 75.4% reported having such structures in place, compared to 9.8% of nonmagnet-seeking organizations (*p*=0.005). Although there was no significant difference in overall shared governance availability (*p*=0.368), UBCs incorporated into shared governance were significantly more prevalent in magnet-seeking organizations (82.4%) than in nonmagnet-seeking organizations (12.3%) (*p*=0.025). No statistically significant association was observed between the magnet-seeking status and the availability of collective bargaining units, with 72.1% of magnet-seeking managers reporting their presence, compared to 11.1% of managers in nonmagnet-seeking organizations (*p*=0.210). These findings highlight the structural and procedural differences in NPR implementation and governance based on the magnet-seeking status (see Table 5).

### 3.3. NPR Perception and Magnet Status

Managers seeking magnet certification were more likely to agree that NPR should integrate with quality and safety efforts (*p*=0.007). They also reported higher frequencies of nurses speaking up about errors or near misses (*p*=0.017). Despite these differences, no significant variations were observed in perceptions of feedback importance or difficulty implementing NPR processes based on the magnet status. However, the findings reinforce the importance of NPR as a developmental and quality improvement tool (see Table 6).

## 4. Discussion

This study provides valuable insights into NPR practices, perceptions, and prevalence among nurse managers, contributing to a deeper understanding of NPR's role in advancing nursing practice. The findings highlighted the structural and procedural elements of NPR implementation within HMC, including nursing-led committees, shared governance structures, and UBCs. These components are critical to supporting NPR activities and aligning them with organizational goals, such as quality improvement and magnet certification.

The majority of nurse managers agreed that NPR fosters clinical growth from novice to expert levels (94.2%) and enhances professional autonomy and accountability (93%), consistent with previous studies emphasizing NPR's benefits in professional development and quality improvement [15, 20–22]. NPR was widely recognized as a tool for promoting quality and safety (88.3%) and for its integral role in providing constructive feedback. However, challenges were evident, with only 62.7% of participants reporting that providing and receiving peer-to-peer feedback were relatively easy and 50% identifying difficulties in implementing NPR processes due to limited resources. In addition to resource limitations, several organizational and cultural barriers may hinder the consistent implementation of NPR. Staffing constraints, such as high patient-to-nurse ratios and limited time for reflective practice, reduce opportunities for meaningful peer review interactions. Furthermore, inconsistent leadership engagement may result in a lack of role modeling and accountability structures necessary to sustain NPR processes. In hierarchical healthcare systems (common in many Middle Eastern contexts), cultural norms may discourage open feedback or create discomfort in critiquing peers, especially across different levels of seniority. These dynamics can limit the effectiveness of NPR and restrict its perceived value among staff. To overcome these challenges, organizations must foster psychologically safe environments, ensure managerial support, and embed NPR into the organizational culture as a norm rather than an exception. Our findings align with the literature highlighting the need for organizational investment and training to overcome NPR implementation barriers [15, 20]. Magnet-seeking organizations demonstrated a higher prevalence of NPR practices, including the integration of UBCs and nursing-led committees into shared governance structures. These findings align closely with the magnet framework, particularly its core components of transformational leadership and structural empowerment. The presence of nursing-led committees and UBCs in magnet-seeking organizations reflects the structural empowerment that facilitates decentralized decision-making and enhances professional governance. Furthermore, nurse managers who support NPR processes act as transformational leaders by fostering a culture of continuous feedback, accountability, and evidence-based practice. The significant association between the magnet-seeking status and increased nurse participation in NPR highlights how these principles are operationalized through leadership behaviors and structural support systems that enable innovation and clinical excellence. These findings support previous research linking magnet certification efforts to enhanced nursing autonomy, shared decision-making, and quality improvement [20]. For instance, the prevalence of formal NPR processes (76.2%) and shared governance structures (79.1%) underscores the commitment of magnet-seeking organizations to NPR as a developmental and quality assurance tool. However, nonmagnet organizations showed lower engagement, indicating opportunities for further NPR integration and standardization across different settings [15]. When compared with international findings, our results reflect similar trends observed in the United States and European healthcare settings, where NPR is often integrated into magnet-recognized institutions and aligned with shared governance frameworks [9]. However, while NPR practices are widely accepted in Western contexts, variations persist in implementation strategies, the degree of staff participation, and reliance on formal feedback systems. In contrast, many Asian and Middle Eastern settings, including Qatar, are still evolving in their approach to peer feedback mechanisms, which may be influenced by differing healthcare governance models and leadership expectations.

Although NPR was perceived positively, peer-to-peer feedback remains underutilized in annual performance evaluations, where managers continue to serve as the primary feedback providers (76.6%). This underscores the need for better alignment with the ANA guidelines, which advocate for peer-driven feedback to enhance teamwork and professional growth [9, 23, 24]. Furthermore, NPR education was reported as inconsistent, with only 52.9% of managers indicating quarterly or monthly training, suggesting the need for streamlined educational programs to ensure uniform awareness and implementation [15, 25]. To address this gap, organizations should establish mandatory NPR training as part of onboarding and continuing professional development programs for nurse managers and clinical staff. This training should focus on the principles of constructive feedback, ethical review practices, confidentiality, and alignment with magnet and ANA guidelines. Additionally, simulation-based workshops, peer mentoring programs, and online modules could be used to provide flexible, role-specific learning opportunities. On a policy level, healthcare institutions should embed NPR competencies into performance appraisal frameworks and leadership expectations, supported by guidelines that promote standardized NPR implementation across departments. Statistical analysis revealed significant associations between the magnet-seeking status and NPR practices, including the higher prevalence of NPR requirements (91.4%) and participation through peer review committees (*p*=0.041). Magnet-seeking organizations also demonstrated higher frequencies of staff speaking up about errors or near misses (*p*=0.017), reflecting a culture of safety and accountability aligned with magnet principles. These findings are consistent with studies emphasizing the role of the magnet status in fostering a positive practice environment and promoting quality initiatives [9, 26, 27].

The results also revealed no significant differences in perceptions of NPR benefits based on the magnet-seeking status, indicating that factors such as organizational culture, educational initiatives, and individual values may play a more substantial role in shaping these perceptions [15, 28]. However, the higher engagement of magnet-seeking organizations in NPR activities reflects their alignment with magnet standards and commitment to nursing excellence.

### 4.1. Limitations of the Study

While the study achieved a satisfactory response rate, limitations should be acknowledged. The use of a convenience sampling method, while practical for accessing nurse managers across diverse hospital settings, may introduce selection bias, as participants who are more engaged with NPR processes may have been more likely to respond. Additionally, reliance on self-reported data raises the possibility of social desirability bias, where respondents may overreport favorable perceptions or practices of NPR. These biases could inflate the perceived prevalence or positivity of NPR engagement. Moreover, because the study focused exclusively on governmental facilities, the findings may not be generalizable to private, military, or other nongovernmental healthcare settings. Additionally, the cross-sectional design restricts the ability to observe changes or trends over time, limiting causal inferences. Nevertheless, the insights gained from this study can still inform peer review practices in private or nonmagnet institutions, as the core components of NPR (such as shared governance, structured feedback, and professional accountability) are universally applicable across healthcare systems seeking to improve nursing practice and quality outcomes.

Moreover, although the response rate was acceptable for survey research, the absence of data from nonrespondents may introduce nonresponse bias, potentially limiting the representativeness of the findings and affecting generalizability. Additionally, the use of a purely quantitative design limits the ability to capture the nuanced experiences and contextual influences affecting NPR implementation. Future research using qualitative or mixed-method approaches could provide deeper insights into individual and organizational perspective. Lastly, the unequal distribution of participants based on the magnet-seeking status presents another limitation, as the higher proportion of magnet-seeking managers may skew the results. Expanding the study to achieve a balanced sample distribution could provide more comprehensive insights. Furthermore, the definition of the magnet-seeking status may lack specificity, potentially affecting the categorization of organizations and the study's findings.

## 5. Conclusion

This study highlights the importance of NPR in fostering professional development, accountability, and quality improvement in nursing practice. It underscores the role of nursing-led committees, shared governance structures, and UBCs in facilitating NPR activities, particularly in magnet-seeking organizations. However, challenges such as inconsistent NPR education, limited peer-to-peer feedback, and resource constraints must be addressed to optimize NPR implementation.

It is recommended that healthcare organizations should prioritize NPR as a core component of staff development and quality assurance. Future policies should align NPR implementations with ANA guidelines and magnet principles, ensuring structured opportunities for peer-to-peer feedback, education, and shared decision-making. Further research is encouraged to explore NPR practices across diverse settings, focusing on specific NPR types, other nursing roles, and alignment with magnet standards. By addressing the identified gaps, stakeholders can promote NPR culture, ensuring its sustainability and maximizing its impact on nursing excellence and patient care outcomes.

## Figures and Tables

**Table 1:** Demographic of the sample (*n* = 244).

Variable	Categories	Frequency (%)
Gender	Male	152 (62.3)
	Female	92 (37.7)

Age/years	Less than 35	15 (6.1)
	35–45	161 (66.0)
	Above 45	68 (27.9)

Education	BSN	134 (54.9)
	Postgraduate diploma	2 (0.8)
	Master's degree	103 (42.2)
	Doctoral degree	5 (2.0)

Nursing experience (years)	Less than 10 years	13 (5.3)
	10–20 years	161 (66.0)
	21 years and above	70 (28.7)

Nursing management experience (years)	Less than 10 years	177 (72.5)
	10 years and above	67 (27.5)

Facility type	General hospital	83 (34.0)
	Tertiary	82 (33.6)
	Ambulatory	25 (10.5)
	Other facilities	54 (21.9)

Workplace	Inpatient departments	77 (31.6)
	Nursing administration	56 (23.0)
	Ambulatory care units	36 (14.8)
	Emergency departments	30 (12.3)
	Intensive care units	17 (7.0)
	Other departments	28 (11.5)

Current job title	Head nurses' level	143 (58.6)
	Nursing house supervisors' level	35 (14.3)
	Director of a nursing level	33 (13.5)
	Executive director of nurse's level	7 (2.9)

Nursing collective bargaining units	Currently available	203 (83.2)
	Not available	41 (16.8)

Magnet certification	Seeking magnet certification	209 (85.7)
	Not seeking magnet certification	35 (14.3)

**Table 2:** The nursing peer review practices.

Variable	Categories		Frequency (%)
Nursing-led committees'/councils' availability	Available		208 (85.2)
	Not available		36 (14.8)

Shared governance availability	Available		193 (79.1)
	Not available		51 (20.9)

Unit-based council availability	Available in every unit or a few units		231 (94.7)
	Not available		13 (5.3)

NPR formal process and incorporated with shared governance	Yes		186 (76.2)
	No		58 (23.8)

NPR required (the participation level)	Required for all or the same levels as nursing required to participate		233 (91.4)
	Not required		21 (8.6)

NPR is not required (the participation level)	Participation is limited to a few units		78 (32.0)
	Participation is limited to a peer review committee		131 (53.7)
	No participation		35 (14.3)

Organizational education for nurses to provide peer to peer training	Never		52 (21.3)
	Annually		63 (25.8)
	More frequent		129 (52.9)

NPR with the annual performance evaluation process	Feedback conducted by managers	Yes	187 (76.6)
		No	57 (23.4)
	Feedback conducted by peer	Yes	161 (66.0)
		No	83 (34.0)
	Feedback associated with the annual evaluation	Yes	144 (59.0)
		No	100 (41.0)

NPR anonymity	Anonymous		73 (29.9)
	Not anonymous		171 (70.1)

NPR process	Consider the nursing experience level		134 (54.9)
	Nurses give feedback about a practice concern		132 (54.1)
	Limited resources to support NPR		122 (50.0)

**Table 3:** Nursing peer review prevalence.

Variables	Unit level	Organizational level	Both levels
	Frequency (%)	Frequency (%)	Frequency (%)
New evidenced-based practice	72 (29.5)	62 (25.4)	100 (41)
Incident-based/risk-based peer review committee	49 (20.1)	82 (33.6)	103 (42.2)
Nurse-to-nurse handoff/shift report	103 (42.2)	49 (20.1)	79 (32.4)
Peer review clinical ladder advancement	58 (23.8)	82 (33.6)	74 (30.3)
Peer interviewing for nursing roles	65 (26.6)	61 (25.0)	88 (36.1)
Peer competency skill assessment	75 (30.7)	64 (26.2)	88 (36.1)
Peer chart audits	69 (28.3)	57 (23.4)	95 (38.9)
Self-reported case review	87 (35.7)	63 (25.8)	65 (26.6)
Peer-reported case review	79 (32.4)	60 (24.6)	84 (34.4)
Focused individual quality review	52 (21.3)	79 (32.4)	92 (37.7)
Annual performance evaluation by managers	70 (28.7)	53 (21.7)	107 (43.9)
Annual performance evaluation by peers	63 (25.8)	59 (24.2)	96 (39.3)

**Table 4:** NPR perception.

Variables	Percentage and (frequencies)				
Level of agreement of NPR benefits					
Items	Strongly agree	Agree	Neither	Disagree	Strongly disagree
NPR helps advance the clinical practice from novice to expert	42.6 (*n* = 104)	51.6 (*n* = 126)	4.9 (*n* = 12)	0.8 (*n* = 2)	0
NPR leads to growth in professional nursing autonomy and accountability	43.4 (*n* = 106)	49.6 (*n* = 121)	6.6 (*n* = 16)	0.4 (*n* = 1)	0
NPR should be integrated with quality and safety efforts to improve practice	46.3 (*n* = 113)	44.7 (*n* = 109)	8.2 (*n* = 20)	0.4 (*n* = 1)	0.4 (*n* = 1)

**Frequency of reporting healthcare incidents**					
**Items**	**Always**	**Often**	**Sometime**	**Rarely**	**Never**

Frequency of nurses “speaks up” about errors or near misses in your organization. As 80.7% answered nurses “speak up” as yes	25.8 (*n* = 63)	40.2 (*n* = 98)	20.9 (*n* = 51)	10.2 (*n* = 25)	2.9 (*n* = 7)

**Level of feedback importance**					
**Items**	**Extremely**	**Moderately**	**Neutral**	**Slightly**	**Not**

Peer nurse managers-to-nurse managers' feedback	47.5 (*n* = 116)	34 (*n* = 83)	16 (*n* = 39)	1.2 (*n* = 3)	1.2 (*n* = 3)
Providing constructive peer-to-peer feedback in all roles to improve practice	44.3 (*n* = 108)	35.7 (*n* = 87)	17.2 (*n* = 42)	2.9 (*n* = 7)	0

**Level of feedback difficulty**					
**Items**	**Very easy**	**Easy**	**Neutral**	**Difficult**	**Very difficult**

Providing constructive peer-to-peer feedback	14.3 (*n* = 35)	48.4 (*n* = 118)	27.5 (*n* = 67)	9.8 (*n* = 24)	0
Receiving constructive peer-to-peer feedback	13.9 (*n* = 34)	48.8 (*n* = 119)	29.1 (*n* = 71)	7.8 (*n* = 19)	0.4 (*n* = 1)
Constructive peer-to-peer feedback is required for the current organization	15.2 (*n* = 37)	47.5 (*n* = 116)	25.4 (*n* = 62)	26 (10.7)	1.2 (*n* = 3)

**Table 5:** Current NPR process about the magnet status.

Variables		Percentage and frequency		Total percentage and frequency out of total responses	Chi-square exact sig.
		Not seeking magnet	Seeking magnet		
NPR required (the participation level)	Not required	3.3 (*n* = 8)	5.3 (*n* = 13)	8.6 (*n* = 21)	**0.004** ^**∗**^
	Required at all/some levels	11.1 (*n* = 27)	80.3 (*n* = 196)	91.4 (*n* = 223)	

NPR is not required (the level of participation)	Little to no participation	3.7 (*n* = 9)	10.7 (*n* = 26)	14.3 (*n* = 35)	**0.041** ^**∗**^
	Limited to NPR committee members/few units	10.7 (*n* = 26)	75 (*n* = 183)	85.7 (*n* = 209)	

Collective bargaining unit availability	Not available	3.3 (*n* = 8)	13.5 (*n* = 33)	16.8 (*n* = 41)	0.210
	Available	11.1 (*n* = 27)	72.1 (*n* = 176)	83.2 (*n* = 203)	

Nursing-led committees and councils	Not available	4.5 (*n* = 11)	10.2 (*n* = 25)	14.8 (*n* = 36)	**0.005** ^**∗**^
	Available	9.8 (*n* = 24)	75.4 (*n* = 184)	85.2 (*n* = 208)	

Shared governance availability	Not available or being developed	2.5 (*n* = 6)	18.4 (*n* = 45)	20.9 (*n* = 51)	0.368
	In place	11.9 (*n* = 29)	67.2 (*n* = 164)	79.1 (*n* = 193)	

Unit-based councils incorporated with shared governance	Not in every unit/only organizational-wide	2.0 (*n* = 5)	3.3 (*n* = 8)	5.3 (*n* = 13)	**0.025** ^**∗**^
	UBC exists in every unit or a few units	12.3 (*n* = 30)	82.4 (*n* = 201)	94.7 (*n* = 231)	

^∗^Significant *p* value less than 0.05 are presented in bold.

**Table 6:** Overall NPR perception and the magnet status.

Variables		Percentages and frequencies out of total responses		Chi-square asym *p*. significance
		Not seeking magnet	Seeking magnet	
*Level of agreement regarding NPR benefits*				
NPR helps advance the clinical practice from novice to expert	Strongly disagree	0	0	0.534
	Disagree	0.4 (*n* = 1)	0.4 (*n* = 1)	
	Neither	0.8 (*n* = 2)	4.1 (*n* = 10)	
	Agree	7.4 (*n* = 18)	44.3(*n* = 105)	
	Strongly agree	5.7 (*n* = 17)	36.9 (*n* = 90)	
NPR leads to growth in professional nursing autonomy and accountability	Strongly disagree	0	0	0.455
	Disagree	0	0.4 (*n* = 1)	
	Neither	1.6 (*n* = 4)	4.9 (*n* = 12)	
	Agree	7.8 (*n* = 19)	41.8(*n* = 102)	
	Strongly agree	4.9 (*n* = 12)	38.5 (*n* = 94)	
NPR should be integrated with quality and safety efforts to improve practice	Strongly disagree	0	0.4 (*n* = 1)	**0.007** ^**∗**^
	Disagree	0.4 (*n* = 1)	0	
	Neither	2.9 (*n* = 7)	5.3 (*n* = 13)	
	Agree	5.7 (*n* = 14)	38.9 (*n* = 95)	
	Strongly agree	5.3 (*n* = 13)	41 (*n* = 100)	

*Reporting healthcare incidents*				
Nurses “speak up” about errors or near misses in your organization	No	4.9 (*n* = 12)	14.3 (*n* = 35)	**0.017** ^**∗**^ (exact *p*)
	Yes	9.4 (*n* = 23)	71.3 (*n* = 174)	

*The frequency of reporting healthcare incidents*				
Frequency of nurses “speaks up” about errors or near misses in your organization. As 80.7% answered nurses “speak up” as yes	Never	1.6 (*n* = 4)	1.2 (*n* = 3)	**0.017** ^**∗**^
	Rarely	2.0 (*n* = 5)	8.2 (*n* = 20)	
	Sometimes	2.5 (*n* = 6)	18.4 (*n* = 45)	
	Often	5.3 (*n* = 13)	34.8 (*n* = 85)	
	Always	2.9 (*n* = 7)	23 (*n* = 56)	

*Level of agreement regarding feedback importance*				
Peer nurse managers-to-nurse managers' feedback	Not important	0.4 (*n* = 1)	0.8 (*n* = 2)	0.662
	Slightly important	0.4 (*n* = 1)	0.8 (*n* = 2)	
	Neutral	2.5 (*n* = 6)	13.5 (*n* = 33)	
	Moderately important	5.3 (*n* = 13)	28.7 (*n* = 70)	
	Extremely important	5.7 (*n* = 14)	41.8 (*n* = 102)	
Providing constructive peer-to-peer feedback in all roles to improve practice	Not important	0	0	0.560
	Slightly important	0.4 (*n* = 1)	2.5 (*n* = 6)	
	Neutral	2.5 (*n* = 6)	14.8 (*n* = 36)	
	Moderately important	3.7 (*n* = 9)	32 (*n* = 78)	
	Extremely important	7.8 (*n* = 19)	36.5 (*n* = 89)	

*Level of agreement regarding feedback difficulties*				
Providing constructive peer-to-peer feedback	Very difficult	0	0	0.424
	Difficult	0.4 (*n* = 1)	9.4 (*n* = 23)	
	Neutral	4.5 (*n* = 11)	23 (*n* = 56)	
	Easy	7.8 (*n* = 19)	40.6 (*n* = 99)	
	Very easy	1.6 (*n* = 4)	12.7 (*n* = 31)	
Receiving constructive peer-to-peer feedback	Very difficult	0	0.4 (*n* = 1)	0.768
	Difficult	0.4 (*n* = 1)	7.4 (*n* = 18)	
	Neutral	4.9 (*n* = 12)	24.2 (*n* = 59)	
	Easy	7.0 (*n* = 17)	41.8 (*n* = 102)	
	Very easy	2.0 (*n* = 5)	11.9 (*n* = 29)	
Constructive peer-to-peer feedback is required to implement for the current organization	Very difficult	0.8 (*n* = 2)	0.4 (*n* = 1)	0.063
	Difficult	2.5 (*n* = 6)	8.2 (*n* = 20)	
	Neutral	3.3 (*n* = 8)	22.1 (*n* = 54)	
	Easy	5.7 (*n* = 14)	41.8 (*n* = 102)	
	Very easy	2.0 (*n* = 5)	13.1 (*n* = 32)	

^∗^Significant *p* value less than 0.05 are presented in bold.

## Data Availability

The data that support the findings of this study are available on request from the corresponding author. The data are not publicly available due to privacy or ethical restrictions.
